# Functional Connectivity of the Anterior Cingulate Cortex and the Right Anterior Insula Differentiates between Major Depressive Disorder, Bipolar Disorder and Healthy Controls

**DOI:** 10.3390/biomedicines11061608

**Published:** 2023-06-01

**Authors:** Anna Todeva-Radneva, Sevdalina Kandilarova, Rositsa Paunova, Drozdstoy Stoyanov, Tina Zdravkova, Ronald Sladky

**Affiliations:** 1 Department of Psychiatry and Medical Psychology, Medical University of Plovdiv, 4002 Plovdiv, Bulgaria; 2 Research Institute, Medical University of Plovdiv, 4002 Plovdiv, Bulgaria; 3 Department of Cognition, Emotion, and Methods in Psychology, Faculty of Psychology, University of Vienna, 1010 Vienna, Austria

**Keywords:** anterior cingulate cortex, salience, insula, depression, bipolar disorder, resting-state functional MRI

## Abstract

**Background:** This study aimed to explore possible differences of the whole-brain functional connectivity of the anterior cingulate cortex (ACC) and anterior insula (AI), in a sample of depressed patients with major depressive disorder (MDD), bipolar disorder (BD) and healthy controls (HC). **Methods:** A hundred and three subjects (n_MDD_ = 35, n_BD_ = 25, and n_HC_ = 43) between the ages of eighteen and sixty-five years old underwent functional magnetic resonance imaging. The CONN Toolbox was used to process and analyze the functional connectivity of the ACC and AI. **Results:** The comparison between the patients (MDD/BD) and HC yielded increased resting-state functional connectivity (rsFC) between the ACC and the motor and somatosensory cortices (SSC), superior parietal lobule (SPL), precuneus, and lateral occipital cortex, which was driven by the BD group. In addition, hyperconnectivity between the right AI and the motor and SSC was found in BD, as compared to HC. In MDD, as compared to HC, hyperconnectivity between ACC and SPL and the lateral occipital cortex was found, with no statistical rsFC differences for the AI seed. Compared to BD, the MDD group showed ACC–cerebellum hyperconnectivity and a trend for increased rsFC between the right AI and the bilateral superior frontal cortex. **Conclusions:** Considering the observed hyperconnectivity between the ACC/somatosensory cortex in the patient group, we suggest depression may be related to an impairment of the sensory-discriminative function of the SSC, which results in the phenomenological signature of mental pain in both MDD and BD. These findings suggest that future research should investigate this particular network with respect to motor functions and executive control, as a potential differential diagnostic biomarker for MDD and BD.

## 1. Introduction

Mental disorders have been established to be one of the determinants of the worldwide burden accounting for 4.9% of global disability-adjusted life years [1]. Among the illnesses contributing to this significant percentage are major depressive disorder (MDD) and bipolar disorder (BD). Moreover, these diagnoses also have high frequency in children and adolescents [2], which can disrupt development and, consequently, their mental health as adults [3].

There is a tendency for comorbidity among different mental disorders, e.g., autism spectrum disorders with mood disorders, schizophrenia, obsessive–compulsive disorder, [4], attention-deficit/hyperactivity disorder (ADHD) with BD, substance-use disorders, etc. [5,6], which challenges the therapeutic process. In addition, a symptomatologic overlap is not rare, e.g., approximately 30% of patients with clinical presentation of depression and anxiety actually suffer from BD, and a main characteristic of this case of comorbidity is the higher frequency and longer duration of the depressive episodes [7,8]. Mixed states and sub-threshold affective states (e.g., subsyndromal manic symptoms; hypomanic symptoms within a depressive episode and affective episodes with psychotic features) are also often observed in clinical practice, which further undermines conventional psychiatric nosology, bringing into question the currently existing categorical classification of mental disorders [9,10,11]. 

In more practical terms, the timely differentiation of MDD and BD is crucial not only for the treatment but also for the prognosis, as well as for relapse and disability prevention strategies. The first presentation of both MDD and BD is often a depressive episode, which makes them practically indistinguishable at onset. Additionally, there is a lack of biomarkers for the differential diagnosis between the two disorders. These two factors lead to many iatrogenic complications, such as the well-known switch to a hypomanic or manic episode in bipolar patients when antidepressants (i.e., the first-line treatment for depression) are not combined with mood stabilizing agents. In the search for such possible biomarkers, a vast mount of research on clinical features, genetic [12], molecular, immunological [13], electrophysiological and neuroimaging characteristics have been performed, with no substantial results as of yet. Nevertheless, some promising findings have been published in the area of functional imaging [14].

One important brain network, relevant for biomarker discovery, could be the salience network (SN) with its major nodes: the anterior insula (AI) and the anterior cingulate cortex (ACC). SN has been implicated in numerous affective tasks, as well as in interoceptive processes such as the identification of both internal and external stimuli relevant for homeostasis [15]. This has consequences for social cognition and other higher cognitive functions, given that, e.g., major nodes of the SN have been determined to be involved in the detection of moral information, which suggests a possible coordinating role in moral decision making [16]. There is compelling scientific evidence that the AI may act as a possible “switch” between major brain networks such as the central executive network and the default mode network (DMN), suggesting its role as a key element in cognitive control [17]. For example, oxytocin (a neuropeptide produced by the hypothalamus with a role in childbirth, lactation, reproduction, social bonding, etc.) has been shown to influence the switching of attention from interoceptive cues towards salient external stimuli via enhancement of the right anterior insula responses and its regulation over the posterior insula [18].

The ACC, on the other hand, is not only involved in emotion processing, but its complex connectivity also underlies a conceivable role in action–outcome learning, as well as reward-related memory [19]. Considering this multifaceted capacity, any structural and/or functional aberration of the key nodes of the SN may manifest as a psychopathological construct, which has been demonstrated in numerous studies showing altered connectivity in major psychiatric disorders such as MDD, BD, and schizophrenia [20,21,22].

For example, a transdiagnostic pattern of grey-matter volume reduction in the bilateral AI and the dorsal ACC was demonstrated in both affective and psychotic disorders. Furthermore, this reduction was not correlated or predicted by the pharmacotherapy in any of the groups [23], which means this alteration is a part of the pathophysiology of these mental disorders, and not induced by the therapeutic intervention. Decreased grey-matter volume of the ACC has been shown in patients with MDD as opposed to BD, whereas BD was characterized by a more significant reduction of grey matter in the hippocampus and amygdala [24]. 

In terms of brain connectivity, increased resting-state functional connectivity (rsFC) between the right dorsal AI and the right superior frontal gyrus has been found in depressed patients with BD in comparison with MDD [25]. In addition, the same study showed an increase in the rsFC between the left ventral AI and the left anterior supramarginal gyrus (aSMG) and the left postcentral gyrus in BD, as opposed to healthy controls (HC) and MDD [25]. Interestingly, a decrease in the rsFC between the thalamus and both major SN nodes, the insula and the ACC, has been outlined in MDD as compared to BD and HC, whereas hypoconnectivity in the prefrontal-thalamic-cerebellar and sensorimotor-thalamic circuits has been observed as a common aberration in both affective disorders, compared to HC [26]. 

Recent research has also demonstrated that alteration of the functional connectivity between the bilateral dorsal AI and the left intraparietal lobule (IPL) can distinguish BD from MDD and HC. In addition, an association was found between reduced right AI and left IPL rsFC and impairment in the reactivity to perceived emotions as well as in the enhanced behavioral drive for rewarding events in both MDD and BD patients, compared to HC [27]. All these aberrations may be related to some of the core symptoms of depression such as disruptive mood regulation, ruminations, negative self-reflection, and sleep disturbances.

The aim of this study was to explore possible differences in the whole-brain functional connectivity of two major nodes of the SN, namely ACC and AI, as seed regions in a sample of both patients with BD and MDD in a depressive episode and healthy individuals. We hypothesized that alterations in the rsFC between the ACC and the AI and nodes from the default mode network and the central executive network may be determined as potential differential signatures of mood disorders and, more specifically, between unipolar and bipolar depression.

## 2. Methods and Materials

A hundred and fifteen subjects between the ages of 18 and 65 years old were included in this retrospective study, of whom 12 were excluded after a quality assessment of the neuroimaging data. The final sample was comprised of 103 participants divided into 3 groups: HC (n = 43; m/f = 14/29; mean age 40.25/SD ± 10.73), MDD (n = 35; m/f = 14/21; mean age 40.97/SD ± 10.86), and BD (n = 25; m/f = 9/16; mean age 41.72/SD ± 9.62). Patients were recruited at several psychiatric clinics and outpatient facilities in Bulgaria, whereas the healthy volunteers were recruited from the community. The participants did not receive financial compensation. All procedures in the research protocol were approved by the Medical University of Plovdiv Ethical Committee (R-2172/03.04.2 015; 2/19.04.2018; P-8632/13.11.2020) and complied with the 1964 Helsinki declaration and its later amendments. A written informed consent was obtained from each subject before their enrolment in the study.

Each participant was assessed by a psychiatrist via a general clinical interview and the structured Mini International Neuropsychiatric Interview (MINI 6.0) [28], as well as the Montgomery–Åsberg Depression Rating Scale (MADRS) [29]. For patients, the clinical diagnosis complied with the DSM-IV-TR criteria for MDD or BD and was based on the interview, the available documentation, and on additional information by family members, whenever possible. All patients were in a current depressive episode, of at least a moderate severity, assessed via the MADRS (score >20 points). Patients with other first-axis comorbid mental disorders, such as alcohol or other substance use disorders, anxiety disorders, obsessive compulsive disorder, post-traumatic stress disorder, eating disorders (anorexia and bulimia), dissocial personality disorder, etc. identified with the MINI interview were excluded from the study.

Any past history of or current psychiatric illness was an exclusion criterion for healthy individuals. Further exclusion criteria for both patients and healthy controls were severe somatic diseases, neurodegenerative diseases, malignant neoplasms, dementia, stroke, current and/or recent infectious diseases, history of head trauma with loss of consciousness, current pregnancy, and metal implants, grafts, or therapeutic devices incompatible with the MRI procedure. 

## 3. MRI Scanning Procedure

The scanning procedure was implemented on a 3T MRI system (GE Discovery 750w, General Electric, Boston, MA, USA). First, a high-resolution structural scan (Sag 3D T1 FSPGR) was performed with the following parameters: slice thickness = 1 mm, matrix size = 256 × 256, TE (echo time) = 2.3 ms, TR (repetition time) = 7.2 ms, and flip angle = 12°. Second, a resting-state functional scan using a 2D Echo Planar Imaging (EPI) sequence was obtained, with a slice thickness of 3 mm, matrix size = 64 × 64, TE = 30 ms, TR = 2000 ms, 36 slices, 192 volumes, and flip angle = 90°. Five dummy scans were acquired before each functional scan, and discarded. During the functional series, all subjects were instructed to remain with their eyes closed and to not think of anything in particular.

## 4. Image Processing

The functional data were processed using the CONN Toolbox [30] (http://www.nitrc.org/projects/conn, accessed on 8 March 2023) running on MATLAB R2022a for Windows. The preprocessing stages included realignment and unwarp, slice-timing correction [31], outlier detection (artifact-detection-tool-based), functional segmentation and normalization, structural segmentation and normalization, and spatial smoothing with a 6 mm FWHM Gaussian kernel. Subsequently, denoising was performed with the following confound regressors: white matter, cerebrospinal fluid (five principal components), realignment parameters (+first derivatives), motion scrubbing (using the default settings of the CONN toolbox), and the exclusion of a minor rest onset trend of each scanning session from the time series. 

Then, seed-based connectivity maps for ACC as well as for right and left AI (defined via the coordinates from the CONN toolbox atlas) were computed for every subject and were subsequently used in a second-level analysis in CONN. Cluster-level inferences on the between-group-level parametric statistics were based on random field theory with a voxel threshold *p* < 0.001 (uncorrected) and a cluster threshold *p* < 0.05 (cluster-size family-wise error (FWE) corrected).

## 5. Statistical Analysis

Differences between the study groups in scale variables were assessed using analysis of variance (ANOVA) followed by protected pairwise comparisons among treatment means. An independent sample *t*-test was used for the comparison between the two patient groups. Associations between categorical variables were assessed using an analysis of contingency tables (χ^2^ test). The specialized statistical software IBM SPSS 28.0 was used for the analysis of demographic and clinical data. The level of significance was set to 0.05 for all tests.

## 6. Results

### 6.1. Socio-Demographic and Clinical Characteristics

Patients and healthy controls did not differ in terms of their age and gender distribution but as expected, HC had significantly higher education (Table 1). Expectedly, the scores on the depression scale were much lower in the control group, but did not differ significantly between the two subgroups of depressed patients. In addition, there were no significant differences between the MDD and BD groups in terms of age at onset, illness duration, and episode duration. However, there was a statistically significant variation in the number of previous episodes, with a higher mean for BD patients as compared to MDD.

### 6.2. Functional Connectivity of the ACC Seed

**Figure 1:**
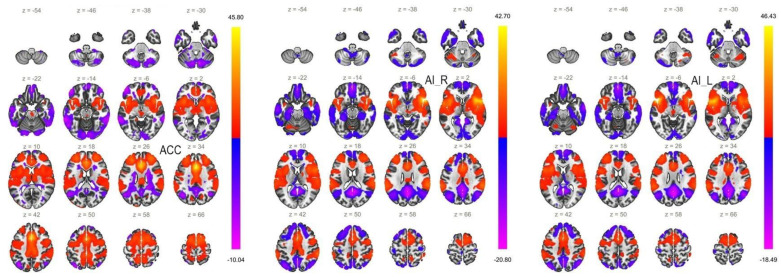
Functional connectivity profile of each seed in all subjects. A cluster threshold of *p* < 0.05 cluster-size-p-FWE correction was applied. As expected, associations were found between each seed and regions of the salience, default-mode, and frontoparietal networks.

The first step of the study was to perform a one-sample *t*-test for all subjects for each seed, so that we could assess the reliability of our findings (Figure 1). As expected, we found associations between each seed and regions of the default mode, salience and frontoparietal networks.

**Figure 2:**
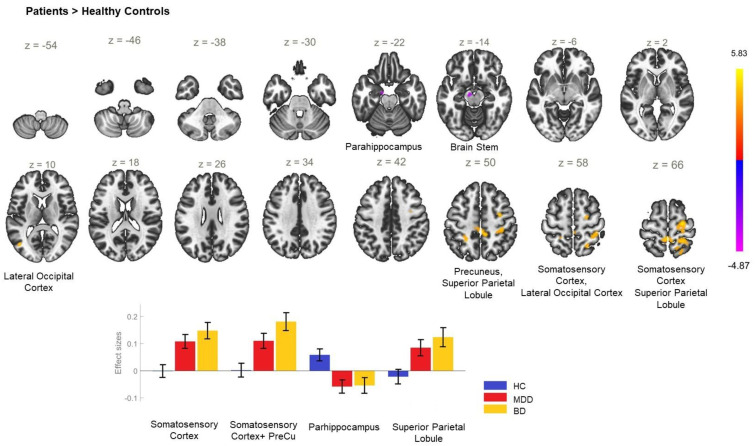
Functional connectivity of the anterior cingulate cortex seed in patients as opposed to the HC group and effect sizes for each significant cluster. Increased resting-state functional connectivity was established between the anterior cingulate cortex and the somatosensory cortex (cluster coordinates +32 −4 +46), extending to the precuneus (cluster coordinates +2 −34 +52), and the superior parietal lobule (cluster coordinates −24 −48 +52), whereas hypoconnectivity was established between the anterior cingulate cortex and the parahippocampal gyrus (cluster coordinates −10 −10 −24) in the patient group, as opposed to healthy controls. Cluster threshold *p* < 0.05 cluster-level FWE correction.

The comparison among all patients (both MDD and BD) and healthy individuals yielded a significantly increased rsFC between the ACC and the three clusters encompassing the right precentral gyrus, bilateral postcentral gyri, bilateral superior parietal lobules, precuneus, and the right lateral occipital cortex, superior division (Table 2). In addition, there was a decreased rsFC between the ACC and the left parahippocampal gyrus, anterior division, as well as between the ACC and the brain stem (Table 2) in the patient group, as opposed to HC. These alterations appeared to be driven by the BD group (Figure 2).

**Figure 3:**
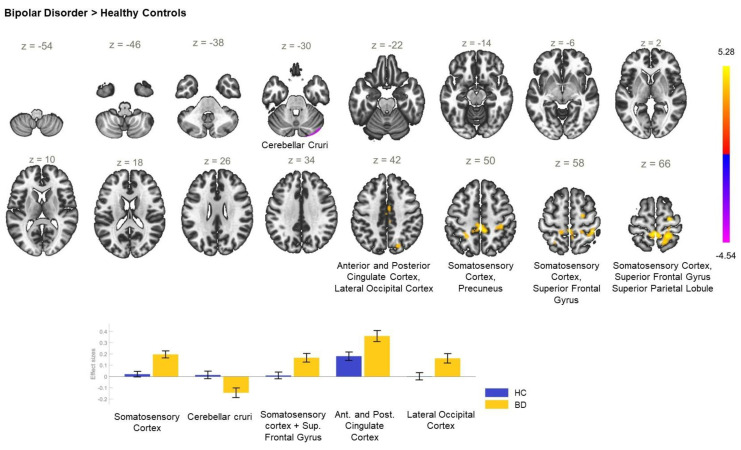
Functional connectivity of the ACC seed in bipolar depression compared to the HC group and effect sizes for each significant cluster with a cluster threshold *p* < 0.05 cluster-level FWE correction. In the bipolar depression group, increased resting-state functional connectivity between the anterior cingulate cortex and the somatosensory cortex (cluster coordinates −2 −36 +66), superior frontal gyrus (cluster coordinates +24 −12 +66), posterior cingulate cortex (−2 −14 +44), and lateral occipital cortex (cluster coordinates +18 −72 +40) as well as reduced resting-state functional connectivity between the anterior cingulate cortex and the right cerebellar crura (cluster coordinates +24 −92 −28), in comparison with HC, were observed.

Accordingly, in BD as compared to HC, the analysis demonstrated a significant increase in the rsFC between the ACC and the right precentral gyrus, the bilateral postcentral gyri, right superior parietal lobule, precuneus, and the superior division of the right lateral occipital cortex in BD, but there was also hyperconnectivity between ACC and the right superior frontal gyrus, posterior cingulate cortex, right supplementary motor cortex, and right cuneal cortex (Table 2). Furthermore, a reduction in the FC between the ACC and both right cerebellar crura (Table 2), was established in BD, as opposed to HC (Figure 3). 

**Figure 4:**
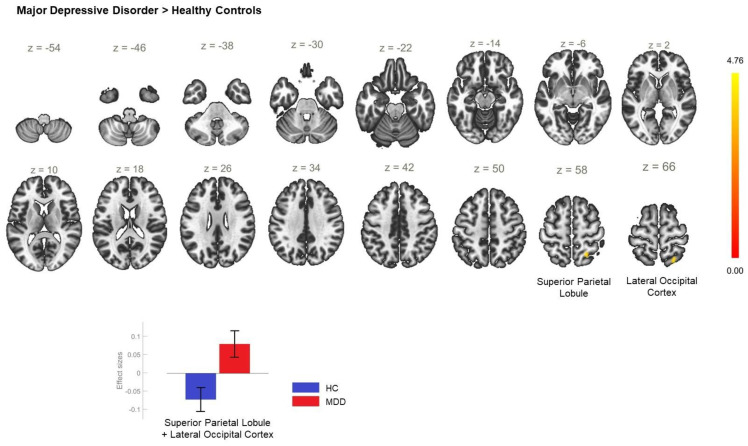
Functional connectivity of the ACC seed in unipolar depression compared to HC and effect sizes for each significant cluster with a cluster threshold of *p* < 0.05 cluster-level FWE correction. Increased resting-state functional connectivity was found between the anterior cingulate cortex and the right superior parietal lobule, as well as the right lateral occipital cortex (cluster coordinates +22 −56 +56) in the major-depressive-disorder group, as opposed to the healthy controls.

**Figure 5:**
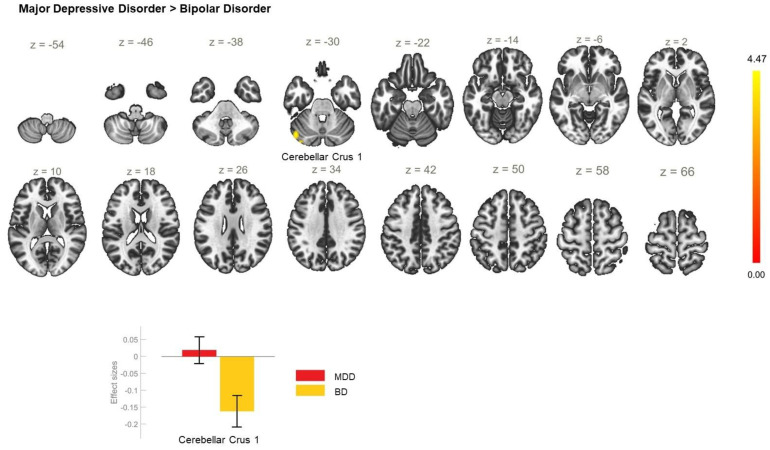
Functional connectivity of the ACC seed in unipolar in comparison with bipolar depression and effect sizes for the significant cluster with a cluster threshold of *p* < 0.05 cluster-level FWE correction. The unipolar-depression group demonstrated increased resting-state functional connectivity between the anterior cingulate cortex and the left cerebellar crus 1 (cluster coordinates −46 −70 −32), which was not observed in bipolar depression.

Enhanced rsFC was also observed between the ACC and the right superior parietal lobule and the right lateral occipital cortex, superior division (Table 2) in MDD, in contrast to HC (Figure 4). The direct comparison between MDD and BD showed hyperconnectivity between the ACC and cerebellum (Table 2) in the MDD group (Figure 5). 

### 6.3. Functional Connectivity of the AI Seeds

**Figure 6:**
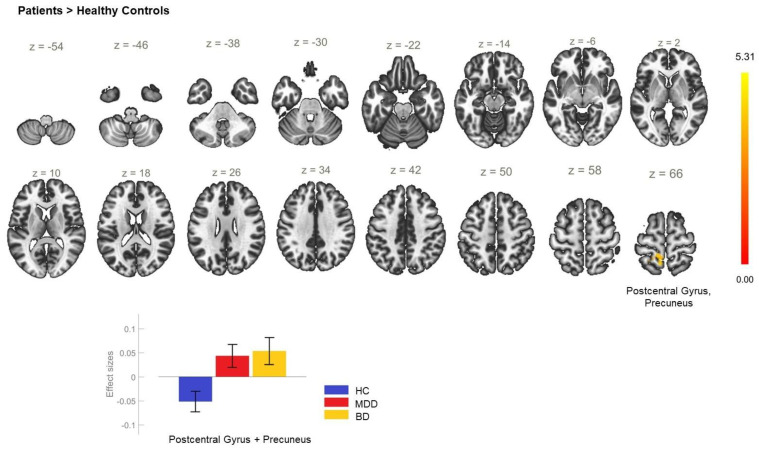
Functional connectivity of the right anterior insula seed in the comparison between the patient and healthy control groups and effect sizes with a cluster threshold of *p* < 0.05 cluster-level FWE correction. There was hyperconnectivity between the right anterior insula and the somatosensory and the precuneus (cluster coordinates −8 −52 +64).

**Figure 7:**
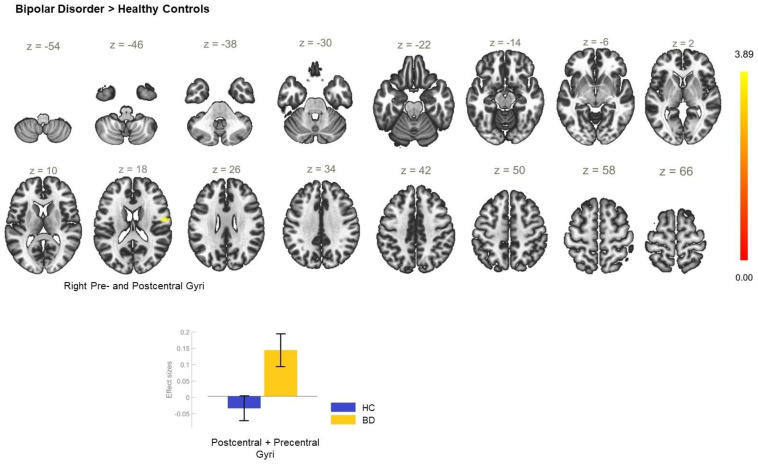
Functional connectivity of the right anterior insula seed and effect sizes with a cluster threshold of *p* < 0.05 cluster-level FWE correction. There was hyperconnectivity between the right anterior insula and the somatosensory and motor cortices (cluster coordinates +54 −6 +18).

**Figure 8:**
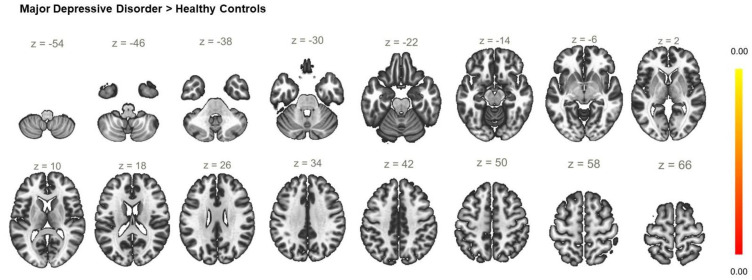
Functional connectivity of the right anterior insula seed with a cluster threshold of *p* < 0.05 cluster-level FWE correction. There were no statistically significant alterations of the resting-state functional connectivity in the MDD group compared to HC.

**Figure 9:**
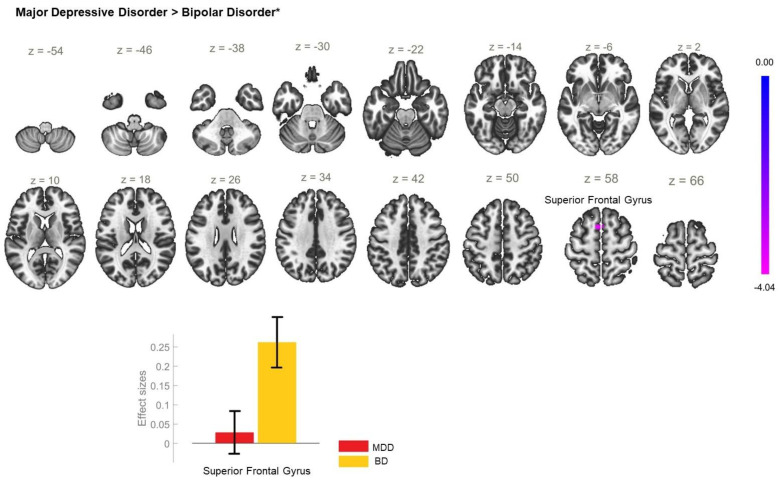
Functional connectivity of the right anterior insula seed and effect sizes with a cluster threshold of *p* < 0.05 cluster-level FWE correction. * The depicted results show a trend, with *p* = 0.068. The MDD group showed a trend for hypoconnectivity between the right anterior insula and the superior frontal gyrus (cluster coordinates −4 +12 +58) in comparison to the BD group.

The analysis of the FC of the left AI seed yielded no significant between-group differences. However, a statistically significant increase in the FC between the right AI and a cluster including left postcentral gyrus, and precuneus (Table 3) was observed in the patient group (Figure 6). When the diagnostic groups were separated, the comparison between BD and HC showed there was statistically significant enhancement of the rsFC between the AI_R and the right pre- and postcentral gyri (Figure 7, Table 3) in BD but there were no differences in the rsFC of the AI_R seed between MDD and HC (Figure 8). The juxtaposition of MDD and BD yielded a trend for hyperconnectivity between AI_R and the bilateral superior frontal gyri; however, it did not reach statistical significance (*p* = 0.068) (Figure 9).

## 7. Discussion

The current study explored the resting-state functional connectivity of two major nodes of the SN, namely the ACC and AI, by means of seed-based whole-brain analysis in patients with depression, in the context of MDD or BD and healthy controls. The first significant finding of this study is the increased rsFC between the ACC and the primary somatosensory and motor cortices, as well as the superior parietal lobule, precuneus, and the right lateral occipital cortex, superior division, in patients with a depressive syndrome in comparison to HC. Furthermore, these alterations seemed to be driven by patients with bipolar depression, as the same regions were significant in the direct comparison of BD vs. HC.

The projections between the ACC and the primary motor cortex have not only been demonstrated to be involved in the initiation and enactment of skilled movements (in humans and monkeys), but also to be under the influence of the prefrontal cortex (PFC) in rhesus monkeys. This suggests a possible controlling function of the PFC on the motor output [32]. However, considering the role of the PFC in cognitive control, we may speculate that aberrant connectivity between the PFC, ACC and the primary motor cortex may be an underlying mechanism for altered executive functions in depression and other mental disorders. In fact, psychomotor agitation and retardation are common phenomenological features of mood disorders which may potentially allow for predicting switches between affective phases and/or the conversion of unipolar to bipolar depression.

Our literature search did not identify research results associated with specific aberrations in the ACC/precentral gyrus connectivity in mood disorders. However, altered activity of the ACC has been implicated in the pathophysiology of BD. For example, a study by King et al. (2018) found a significant reduction in the activity of the ACC during a bilateral finger-tapping task in youths with BD (in euthymia), as compared to HC, which was suggested as a possible impairment of the ACC modulation between emotion and motor processing [33]. Motor impulsivity in BD has also been associated with a decrease in the GMV in the ACC [34]. In tasks involving emotion processing, an increase in ACC activity was observed during manic episodes [35]. Considering these findings, we may suggest altered FC between ACC and the primary motor cortex as a possible feature that differentiates depression in BD from MDD. In addition, the alterations in psychomotor activity have been correlated with latent bipolarity and possible mood switching in patients with MDD [36,37,38]. In sum, these findings suggest that future research should investigate this particular network with respect to motor functions and executive control, as a potential differential diagnostic biomarker for MDD and BD.

The ACC is known to be involved in many functions. Highly relevant for depression is the processing of both physical and psychological pain, where the ACC is relevant for the affective component [39]. The primary somatosensory cortex, on the other hand, is associated with the sensory-discriminative component [39] and increased rsFC between the thalamus and the SSC has been found in MDD, as compared to HC, which correlates with core symptom severity of MDD, such as cognitive dysfunction, anhedonia, etc. [40]. Conversely, an experimental study of rats showed that the connection from the posterior thalamic nucleus to the primary SSC is involved in the mechanisms of neuropathic pain associated with tissue injury, whereas the connection between the parafascicular thalamic nucleus and ACC mediates allodynia in depression-like states [41]. Taking into account our findings of increased rsFC between the ACC and the SSC in the patient group, we suggest that depression may be related to an impairment of the sensory-discriminative function of the SSC, which results in the phenomenological signature of mental pain in both MDD and BD.

A study has demonstrated that intranetwork alterations in the DMN may underpin the classification of two separate MDD biotypes, depending on the hyper- (namely, between the left superior frontal cortex and the precuneus) or hypoconnectivity within the DMN [42]. Furthermore, the within-DMN hypoconnectivity was associated with a poorer treatment response. All this evidence suggests that the functional aberrations of the precuneus may not only be an underlying mechanism for cognitive and sleep disturbances in mood disorders, but also a possible region of interest in the establishment of biomarkers for classification and therapeutic response in MDD and BD. In our study, the BD group was also associated with hyperconnectivity between the ACC and the precuneus. Altered connectivity of both regions, as well as the AI, has been associated with poor sleep quality in depression [43]. Moreover, enhanced rsFC between the right inferior frontal gyrus (IFG) and the precuneus was found in all three types of episodes (namely, mania/hypomania, euthymia, and depression) in BD, in comparison with healthy individuals, and this hyperconnectivity was linked to impaired cognitive performance [44]. Interestingly, increased rsFC between the frontoparietal network and the left precuneus, as well as between the dorsal attention network and the precuneus in pre-treatment bipolar depression was demonstrated to be concordant with a therapeutic response to Lamotrigine [45]. The precuneus has been implicated in the pathophysiology of MDD, as well. 

Another one of the interesting findings in our study was that the rsFC between the ACC and the right superior parietal lobule was increased in both MDD and BD, as compared to HC. The SPL has been implicated in the integration of the somatosensory and visuomotor processes [46], and especially in the simultaneous processing of multiple visual elements [47]. Therefore, ACC-SPL hyperconnectivity may explain the common cognitive symptoms in both disorders—dysfunctional perception, hypoprosexia, and pseudodementia. Rolls et al. have found the same increase in the ACC/SPL connectivity in patients with unipolar depression in comparison to HC, as well as increased rsFC between the ACC and the orbitofrontal cortex, middle and inferior frontal gyri, temporal cortical areas, etc. [48]. Notably, this hyperconnectivity was demonstrated in unmedicated patients but not in medicated ones. However, all participants in our study were on stable medication, and this alteration was observed both in patients with MDD and those with BD. Yet this discrepancy may be explained by the insufficient pharmacodynamic effects of the medications. If this finding is confirmed on a larger sample, and in another direct comparison between medicated and unmedicated patients, it may not only suggest a common etiopathophysiological mechanism of the cognitive disturbances in both disorders, but also a possible target for assessment of the therapeutic outcome.

A compelling finding in the study turned out to be the reduction of the rsFC between the ACC and the cerebellum in BD as opposed to HC, and between ACC and the left cerebellar crura in BD compared to MDD. The literature shows that dysfunction of the cerebellar metabolism has been found in MDD, as described in a meta-analysis by Liang et al. [49]. Moreover, in unmedicated BD type II, impaired rsFC was demonstrated between the right cerebellar crus 1 and the PreCu and the left crus 2 and the medial prefrontal cortex, as compared to HC [50]. The cerebellum has been implicated in the processing of psychological pain and, considering the significant association between psychological and physical pain as well as between psychological pain and suicide risk in depression [51], alterations in its connectivity may well be not only a future diagnostic marker but also a therapeutic target. Of note is the fact that cerebellar dysfunctions are also relevant to psychomotor regulation, which is theoretically associated with the increased connectivity between the ACC and the primary motor cortex.

The lateralization of brain function remains one of the most interesting areas of research, especially since it has been demonstrated in both vertebrate and invertebrate species [52]. In our study, alterations in the rsFC of the right AI have demonstrated significant between-group differences, unlike the left AI, raising the question about the functional specificity of the right AI as a hub of interest in the pathophysiology of mood disorders. Recent scientific evidence suggests a major role for the right AI in interoceptive processes [53,54]. Moreover, the right AI has been associated with the affective-perceptual form of empathy, whereas the left AI has been determined to be involved in both the cognitive-evaluative and affective-perceptual forms [55]. These findings, combined with the current results of our research confirming alterations in the connectome between the right AI, somatosensory and motor cortices in patients with BD, imply a disturbance in the interoceptive and affective integration in BD in comparison with HC. Furthermore, the hypoconnectivity between the right AI and the right MFG, compared to the left AI, may be interpreted as an impairment in both interoception and cognitive control in MDD as opposed to healthy individuals, which suggests the possibility of a divergent degree of dysconnectivity across a continuum with two phenomenological substrates, namely, MDD and BD.

The current study has some limitations that should be considered. Firstly, the sample size, although substantial as a whole (n = 115 subjects), is limited in terms of comparison groups and a future expansion of it may uncover new findings for MDD and BD. Secondly, this is a cross-sectional study, and the observed alterations may vary in the longitudinal course of illness, which is grounds for future research on the matter. And last but not least, all our patients were on stable pharmacotherapy, due to clinical and ethical considerations, which may have introduced a certain bias into the results. Therefore, we interpreted them with great caution and tried to put our results in the context of previous studies where unmedicated patients were recruited. It should be considered, however, that most of the mainstream research in psychiatric neuroimaging shares the same limitations [56].

## 8. Conclusions

Our research adds to the growing evidence of the involvement of the salience network in the pathophysiology of mood disorders. Furthermore, the observed alterations may explain some of the cognitive, psychomotor, emotional, and volitional disturbances that are observed in the phenomenology of both MDD and BD. Additionally, our findings suggest both common and distinct aberrations in unipolar and bipolar depression, which may be viewed as supporting the hypothesis of an existing continuum of mood disorders.

## Figures and Tables

**Table 1:** Socio-demographic and clinical characteristics of the sample.

	HC n = 43	MDD n = 35	BD n = 25	Significance Level between MDD and BD	Significance Level between HC and MDD & BD
Age (mean ± SD)	40.25 (±10.73)	40.97 (±10.86)	41.72 (±9.62)	*p* = 1.000 ^c^	*p* = 1.000 ^a^/1.000 ^a^
Gender (M/F)	43 (14/29)	35 (14/21)	25 (9/16)		*p* = 0.793 ^b^
Education (years)	15.93 (±3.26)	13.8 (±2.93)	13.88 (±2.71)	*p* = 0.915 ^c^	*p* = 0.012 ^a^/0.035 ^a^
MADRS	2.28 (±2.25)	30.2 (±4.14)	29.76 (±6.17)	*p* = 0.742 ^c^	*p* < 0.001 ^a^/0.001 ^a^
Age at onset (mean ± SD)	-	30.24 (11.01)	29.72 (9.19)	*p* = 0.850 ^c^	-
Illness duration (mean ± SD)	-	130.97 (113.93)	148.6 (101.64)	*p* = 0.544 ^c^	-
Episode duration (mean ± SD)	-	21 (36.65)	15.8 (14.99)	*p* = 0.508 ^c^	-
Number of previous episodes (mean ± SD)	-	3.97 (3.56)	6.36 (4.72)	*p* = 0.035 ^c^	-

^a^—ANOVA; ^b^—χ^2^ test; ^c^—Independent samples *t*-test.

**Table 2:** Between-group differences of functional connectivity of the ACC seed.

Between-Group Contrast	MNI Coordinates x, y, z	Cluster Size	Cluster Threshold (*p* < 0.05, FWE)	Regions within the Cluster
P > HC	+32 −4 +46	697	<0.001	PostCG_R, PreCG_R, SPL_R
	+2 −34 +52	526	<0.001	PostCG_R, SPL_R, PreCu, LOC_R—superior division, PostCG_L
	−24 −48 +52	106	0.017	SPL_L
HC > P	−10 −10 −24	109	0.015	PHPG_L—anterior division, Brain stem
BD > HC	−02 −36 +66	1393	<0.001	PostCG_R, SPL_R, PostCG_L, PreCu
	+24 −12 +66	120	0.009	PreCG_R, SFG_R
	−2 −14 +44	105	0.018	ACC, PCC, SMC_R
	+18 −72 +40	86	0.047	LOC_R—superior division, PreCu, CC_R
HC > BD	+24 −92 −28	138	0.004	CerC1_R, CerC2_R
MDD > HC	+22 −56 +56	112	0.013	SPL_R, LOC_R, superior division
MDD > BD	−46 −70 −32	191	<0.001	CerC1_L

PostCG_R/L—Right/Left Postcentral Gyrus; PreCG_R/L—Right/Left Precentral Gyrus; SPL_R/L—Right/Left Superior Parietal Lobule; LOC_R, superior division—Right Lateral Occipital Cortex, superior division; PHPG_L, anterior division—Left Parahippocampal Gyrus, anterior division; SPL_L—Left Superior Parietal Lobule; PreCu—Precuneus Cortex; CerC1_R/L—Right/Left Cerebellum Crus1; CerC2_R—Right Cerebellum Crus2; SFG_R—Right Superior Frontal Gyrus; ACC—Cingulate Gyrus, anterior division; PCC—Cingulate Gyrus, posterior division; SMC_R—Right Supplementary Motor Cortex; CC_R—Right Cuneal Cortex; SFG_R/L—Right/Left Superior Frontal Gyrus.

**Table 3:** Between-group differences of functional connectivity of the right AI seed.

Between-Group Contrast	MNI Coordinates x, y, z	Cluster Size	Cluster Threshold (*p* < 0.05, FWE)	Regions within the Clusters
P > HC	−8 −52 +64	201	<0.001	PostCG_L, PreCu
BD > HC	+54 −06 +18	108	0.017	PostCG_R, PreCG_R
MDD > BD	−4 +12 +58	80	0.068	SFG_L, SFG_R, SMC_R

PostCG_R/L—Right/Left Postcentral Gyrus; PreCG_R—Right Precentral Gyrus; PreCu—Precuneus Cortex; SMC_R—Right Supplementary Motor Cortex; SFG_R/L—Right/Left Superior Frontal Gyrus.

## Data Availability

All data is available from the authors upon reasonable request.
